# Defining the elements of a self and peer assessment system for academic institutions of public health in Africa as a precursor to accreditation: A study protocol

**DOI:** 10.1371/journal.pone.0334645

**Published:** 2025-10-21

**Authors:** Neo R. T. Ledibane, Sean M. Patrick, Kuku V. Voyi

**Affiliations:** School of Health Systems and Public Health, Faculty of Health Sciences, University of Pretoria, Pretoria, Gauteng Province, South Africa; University of the Witwatersrand, SOUTH AFRICA

## Abstract

**Background:**

Current challenges facing the overburdened health systems in Africa, warrant a review of the public health training and development of the health workforce for the attainment of the envisioned global goal of universal health coverage (UHC) and the United Nations Sustainable Development Goals (SDG). The integral components informing the relevance of public health education in the context of UHC comprise the academic workforce, curricula and the capacity of the academic institutions of public health. This study aims to assess the quality improvement strategies of academic institutions of public health with respect to the curriculum and the academic faculty staff within the WHO African region, in order to develop an institutional self- and peer-assessment tool to ensure the quality of public health education.

**Methods:**

This study will be a three-phase, multicentre sequential mixed-methods design within the WHO African region, targeting 52 ASPHA (Association of Schools of Public Health in Africa)-affiliated institutions. Phase 1 will comprise a cross-sectional survey to determine the current programme assessment standards. Phase 2 will employ a modified Delphi method to reach consensus on these standards that will comprise a newly developed assessment tool. Phase 3 will pilot the tool through institutional self-assessment. MPH coordinators and department heads will participate. Convenience sampling and electronic questionnaires will be used. Quantitative data will be analysed using STATA 18, and ATLAS.ti for qualitative data.

**Projected outputs and impact:**

The study findings are envisaged to result in a set of agreed standards reflecting the institutional arrangements to ensure quality postgraduate public health education in Africa. These standards, in the absence of regulation or formal accreditation, will be the basis of a self- and peer-assessment tool to enable the African academic institutions of public health to advance capacity development and monitor progressive educational goals suitable for local health needs, within the global context of the SDG and UHC implementation. Thus, laying the ground for a uniquely African accreditation system. The findings will be shared with the relevant stakeholders; ASPHA, the academic institutions of public health, and the scientific community at conferences and published in accredited journals.

## Introduction

The 2030 Agenda for Sustainable Development, adopted by all United Nations Member States in 2015, provides the common blueprint for health as defined by the World Health Organization (WHO). According to the WHO, health is defined as “a state of complete physical, mental and social well-being and not merely the absence of disease or infirmity” [1]. The current United Nations (UN) Sustainable Development Goals (SDG) serve as a global partnership for a single health goal to, “Ensure healthy lives and promote well-being for all at all ages” [2]. Despite the continuing challenges (including major communicable and non-communicable diseases, poor access to essential health services, and major financial constraints), notable progress has been made with overall reduction in morbidity and mortality, and increased life expectancy. Concerted efforts by all the relevant stakeholders are required to address the continuing challenges to achieve Universal Health Coverage (UHC) – meaning that people will receive health services they need without suffering financial hardship [1–3].

Therefore, a well-trained public health workforce in adequate numbers is essential to provide integrated, people-centred health services to meet the population health needs, and is thus central to the achievement of UHC and of SDG3 (which aims to ensure healthy lives and promote well-being for all at all ages) [2]. This being a central component of the global and national Human Resources for Health (HRH) strategies [3,4]. Although the HRH strategy highlights the need to upscale the number of public health professionals, the African health systems remain burdened and curatively-oriented [3,5–7]. Therefore, the training of such a workforce needs to be reviewed and evaluated regularly to ensure good practice and quality education [8–13].

### Public health competencies

The core disciplines of public health, epidemiology and biostatistics, are cross-cutting and provide the tools for describing the patterns and trends, finding causal linkages and evaluating the effectiveness of health services and programmes [8,10,14,15]. The assessment of how well the current education training frameworks are responsive to (and linked with) major societal and scientific shifts involves macro trends. These are defined as a set of competencies and recommendations for epidemiologic and public health training to address the changing landscape. A competency is defined as a cluster of related knowledge, attitudes, and skills which are important for the performance of a job activity and can be measured against well-accepted standards [8,9,16,17].

Standards are levels of quality or attainment, norms or model in comparative evaluations [9]. Macro trends are often considered in strategic planning processes and involve some combination of changing demographics, economic factors, technological changes, and legal, political, or social conditions [10,16]. Public health practitioners should demonstrate skills and competencies in leadership, systems thinking, policy development, critical and analytical thinking, as well as teamwork and communication skills in order to improve the performance of the health system and population health outcomes [18–21]. While some regions such as Africa, are starting to focus on globally relevant, region-specific core competencies [22], other regions (Europe and North America) are already advanced [18,23,24]. Therefore the developing regions have an opportunity to learn from those that have already pioneered in this regard [25].

### Public health workforce capacity and training in Africa

The training and development of the health workforce, including the public health workforce, is vital to their productivity, performance, distribution, and retention; and the selection and education of the health workforce is closely related to the broader social and economic development [3,26]. As one of the building blocks of health system strengthening, the health workforce impacts the overall health goals and health outcomes [27,28]. The various strategies and commissions which reiterate the need for approaches trailblazer and partnerships to close the existing sectoral gaps include, the Global Strategy on Human Resources for Health [29], the report of the Commission on Health Employment and Economic Growth [26]; and the Lancet Commission on transformative learning and education [21]. The pursuit of these being to enable the scaling up of transformative, quality education and fostering life-long learning. Additionally, they emphasise the reforms which are necessary for health workforce competencies that are best suited to equitably meet the population needs and health labour market needs for strengthened health systems [21,30].

The current challenges in public health reform are partly attributable to the non-responsive postgraduate public health education curricula in relation to the quantity and pertinence of training which are not appropriately aligned with the population health needs [5,13,29]. The corresponding correction is required to envisage the strengthening of health systems in order to improve health service provision and health outcomes, which require highly skilled public health personnel with expertise and knowledge [27].

### Public health training: Role of academic institutions in Africa

The role of the academic institutions in training health professionals in the discipline of public health is crucial to ensure the successful implementation of the UHC [31]. Public health education and training takes place at undergraduate and postgraduate levels. It is most often included in the undergraduate health disciplines such as medicine or dentistry; although not exclusively [32]. Postgraduate programmes include: the Master of Public Health (MPH) which targets professionals from varied health and social science disciplines; and a medical specialisation only for medical doctors, the Master of Medicine in Public Health Medicine (MMed PHM) [32,33].

The main aim of the programmes is to equip health professionals from a variety of disciplines with key public health competencies and collaborative strategies to address population level risk factors contributing to the national and global burden of disease [5,7]. In addition, the programmes equip practitioners to become pioneering and innovative professionals with an emphasis on multidisciplinary approaches that utilise the advanced evidence-based scientific knowledge [5–7]. Postgraduate programmes in public health are generally recognised professional postgraduate qualifications for leadership positions within the health sector [5,34–36].

### Licensing and accreditation

Licencing (or licensure) is a process by which governmental authority grants permission to an individual or organisation to operate or engage in an occupation [24,37]. These regulatory processes include the establishment of education standards, quality assurance of education programmes, establishment of codes of conduct, identification of scopes of practice, systems for licensure, maintenance of registers of those fit to practice, and systems to ensure continuing professional development and appropriate disciplinary measures. Many of these attributes are determined by the interaction between the market forces, political benefits, and health workforce regulation [38–40].

An accreditation system is an essential element to determine whether a programme meets minimum quality standards [41]. Accreditation in higher education is a voluntary process which is based on self- and peer-assessment for the purpose of assuring and improving education quality and accountability [42].

Examples of accreditation systems in established to monitor and assure the quality of public health capacity and education include, the Council on Education for Public Health (CEPH) in the United States of America (USA) [42]; *Ahpra*, a Canadian regulatory agency; the Health and Care Professions Council (HCPC) in the United Kingdom (UK Faculty of Public Health or Pubic Health England); European Agency for Accreditation in Public Health Education (APHEA) in Europe and the National Registration and Accreditation Scheme in Australia [24,25,41].

However, no such system exists in the African region. A South African study highlighted the desire expressed by the MPH coordinators for the need of an association or quality assurance body for the establishment of quality standards to ensure benchmarking and standardization across the various programmes [5].

Although the cumulative number of MPH graduates over a 5-year period can be considerable exceeding 800 for one country [5], it is concerning to note that the programme is not regulated by any council, or accredited by any professional body in Africa. The capacities of the African academic institutions, to assess their own capacity to implement the training of the necessary health workforce in response to UHC, cannot be clearly determined due to the lack of the established standards of public health education, practice or regulatory framework [25,38,43].

### Rationale for the study

#### Problem statement.

The practice of public health is becoming more prominent globally, particularly in low-to-middle income (LMIC) countries, as a result of constrained financial and human resources amidst a disproportionate burden of disease [33]. Most of the countries in sub-Sahara Africa (SSA) face a mixed burden of disease [44]; and subsequently, public health (also known as, public health medicine, community medicine, or preventive medicine) has been identified as a strategic approach by some of the National Ministries/ Departments of health to reduce the burden of disease and reduce dependence on hospital-based and specialist services [33].

The current challenges, as well as the need to address both the SDGs and UHC within the context of overburdened health systems warrant a review of public health education within the African region. There is an urgent need for evidence to enable informed decision-making to ensure proper alignment with the production and development of the public health workforce, who should be trained to derive fitting solutions for the African health challenges [11,12,22].

This study is thus in response to and aligned with the six of the seven resolutions undertaken at the 2019 ASPHA (Association of Schools of Public Health in Africa) held in Kampala, Uganda; to support a sustainable health workforce development initiative by increasing the quantity and the quality of the public health workforce in Africa [45]. This research will focus on the institutional capacity, including the academic workforce and curriculum renewal processes of academic institutions of public health in Africa, but will not revise curriculum content as that is the focus of another project by Opare et al [11].

The Association of Schools of Public Health in Africa (ASPHA) mainly functions as a forum to serve the collective needs of the member institutions in the education and training of public health professionals by building capacity to maximize and excel in academic training and advocacy for public health policy. Additionally, the association serves as a medium for sharing innovative ideas to advance scientific knowledge led by the regional experts, as well as to provide a strong unified voice for promoting regional public health development and practices in Africa [17].

The integral components of the ASPHA projects will determine the relevance of public health education in the African region and comprise the curriculum (process), the academic workforce (people), and the academic institutions of public health (places). Thus, the results of this research will contribute to pursuit of developing an accreditation system in the region and professionalisation of the field. This shift towards professionalisation of public health has been reiterated by Mansholt et al. at the European Union Public Health Association (EUPHA) Health Workforce Research Conference held in June 2020, as well as the 16^th^ World Congress on Public Health (WCPH) held in October 2020 [25,46,47]. The emphasis is that the health professional regulatory processes remain central to ensuring health workforce quality and sustainability to address priority health systems concerns, as well as associated capacity constraints, which the COVID-19 pandemic has highlighted [40].

### Significance of the research

The significance of the proposed research is to establish the first tools and processes needed for an accreditation system for academic institutions of public health in Africa. It is envisaged that the outcomes of this research will be of critical academic and practical value to the academic institutions, as well as to the region and professionalisation of public health as a whole. Furthermore, this research could provide essential information that can guide academic institutions of public health in strengthening their capacity and collaboration. Regional and global collaborations have the potential to foster partnerships, as well as regional public health workforce development [48,49].

The absence of regulatory bodies for public health can be overcome by a voluntary system of accreditation, which has proven to be very successful in establishing and maintaining the capacity of institutions to provide quality public health education in other regions such as Europe and North America [24,42]. This research will therefore focus on determining the elements needed for self- and peer assessment which is the hallmark of accreditation systems. Given the varied country experiences in health professional regulation and the absence of a regional accreditation system, it will be essential to understand what works best for differing settings, and how to maintain and improve the quality of the public health education, in particular the MPH programme [39,47].

### Research questions

What is the current capacity status of academic institutions of public health in Africa, as it relates to the academic workforce (people), the curriculum (process) and the academic institutions (place)?

Are the African academic institutions of public health able to prepare MPH graduates for UHC?

## Aim and objectives

### Study aim

The aim of the study is to assess the quality improvement strategies of academic institutions of public health with respect to the curriculum and the academic faculty staff within the WHO African region, in order to develop an institutional self- and peer-assessment tool to ensure the quality of public health education.

## Study objectives

### PHASE 1: Exploration of the current status of the academic institutions of public health in Africa

**Objective 1:** To describe the internal strategies for quality assurance of the teaching, learning methods/practices and curriculum renewal

**Objective 2:** To describe the characteristics of academic staff and (internal) strategies used to develop academic staff.

**Objective 3:** To determine the interest and need of an accreditation system for academic institutions of public health.

### PHASE 2: Development of standards and assessment tool

**Objective 4:** To develop public health teaching and learning quality standards.

**Objective 5:** To develop an institutional self- and peer-assessment tool to monitor and evaluate progress and ensure continual adherence to the public health teaching and learning quality standards.

### PHASE 3: Piloting of the developed tool

**Objective 6:** To pilot the newly developed self- and peer-assessment tool for public health education in Africa through institutional self-assessment and develop a recommended implementation framework.

### Methods

**Fig 1:**
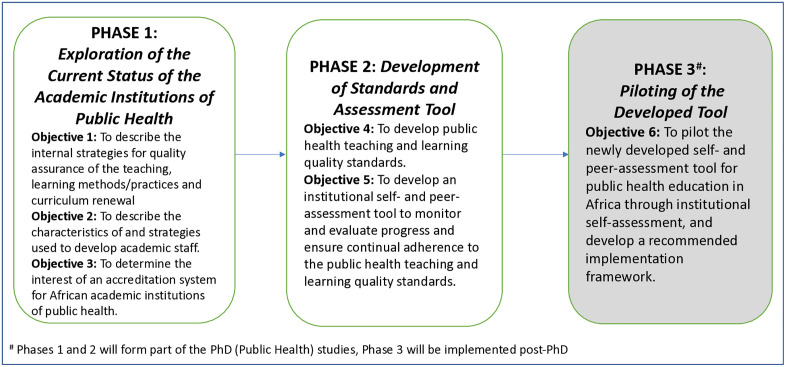
Diagram illustrating the various phases and objectives of the study.

This study will be a three-phase, multicentre embedded sequential mixed-methods design [50] within the WHO African region, targeting 52 ASPHA (Association of Schools of Public Health in Africa)-affiliated institutions (Fig 1). The quantitative component will include an analytical cross-sectional study design to assess the relationships between different study variables, and the qualitative data analysis of open-ended questions will include thematic analysis of the emerging themes and sub-themes [14,51].

PHASE 1 is currently ongoing and will conclude at the end of May 2025. This cross-sectional online survey (quantitative/ qualitative) will ascertain the current status of the academic institutions of public health and to determine which items should be included as standards for the assessment tool. PHASE 2, will commence from August 2025 until September 2025, and will comprise a modified Delphi method (qualitative) to reach consensus on the standards to be included in the tool. PHASE 3, (post-PhD ~ 2026/ 2027) will comprise the piloting of the tool by willing institutions (quantitative/ qualitative) and to recommend the implementation framework.

The following components will be common for the first two phases: study setting and study population/participants.

### Study setting

The study setting will be at the 52 ASPHA-affiliated academic institutions of public health in the Anglophone countries (where English is the official language), the Lusophone countries (where Portuguese is the official language) and the Francophone countries (where French is the official language) in the WHO African Region (Table 1) [52].

**Table 1:** Number of ASPHA-affiliated academic institutions of public health in Africa by sub-region and language [17].

SUB-REGION	NUMBER OF ACADEMIC PUBLIC HEALTH INSTITUTIONS PER COUNTRY	TOTAL NUMBER OF PH INSTITUTIONS
**NORTHERN AFRICA**	**Tunisia (1; English)**	**1**
**WESTERN AFRICA**	**Burkina Faso (1; French)** **Ghana (4; English)** **Nigeria (14; English)**	**19**
**CENTRAL AFRICA**	**Democratic Republic du Congo (1; French)**	**1**
**EASTERN AFRICA**	**Ethiopia (4; English)** **Kenya (9; English)** **Malawi (1; English)** **Mocambique (1; Portuguese)** **Somalia (2; English)** **South Sudan (1; English)** **Tanzania (1; English)** **Uganda (2; English)** **Zambia (1; English)**	**22**
**SOUTHERN AFRICA**	**Botswana (1; English)** **Namibia (1; English)** **South Africa (7; English)**	**9**
**TOTAL**		**52**

### Study Population and Participants

**Fig 2:**
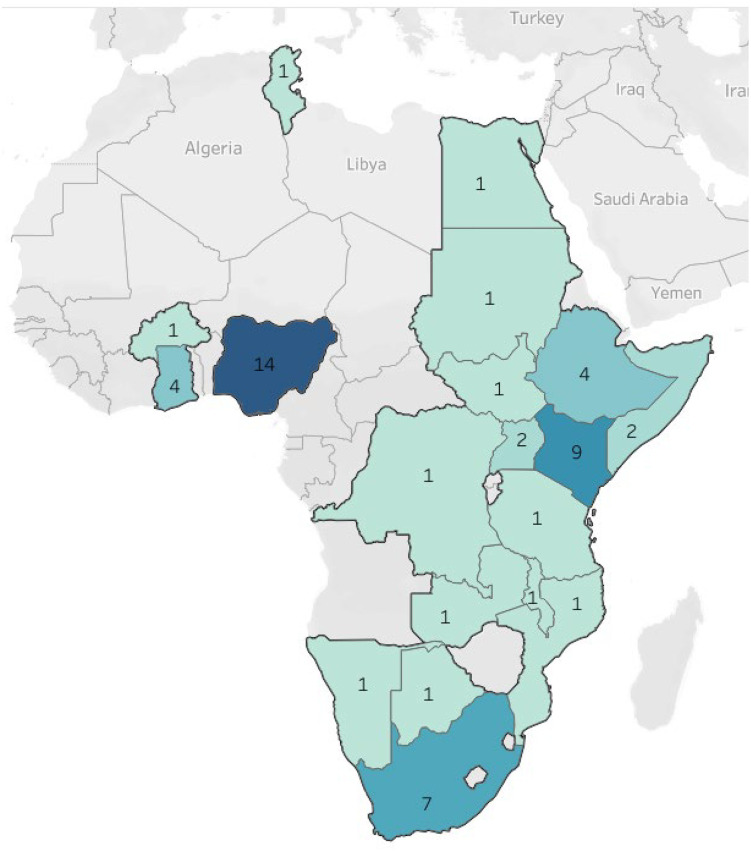
A map showing the 52 ASPHA member institutions across 19 African countries and the number of academic institutions per country (Reprinted from ASPHA under a CC BY license, with permission from ASPHA, original copyright 2024) (Fig2.tif).

The study population and participants comprise the MPH programme coordinators at 52 public health academic institutions across 19 countries affiliated with the Association of Schools of Public Health in Africa (ASPHA), and recruited through the ASPHA (Fig 2) [14].

**Phase 1: Exploration of the current status of the academic institutions of public health**

**Study design:** An analytical cross-sectional study design will be used for Phase 1 [14].

**Methodology:** The total population sampling method will be used where all the 52 academic institutions of public health affiliated with ASPHA will be recruited to participate [14,53]. A list of the academic institutions of public health will be obtained from the ASPHA database [17]. All the institutions will be forwarded an email to explain the nature and purpose of the study and to obtain consent before taking part.

**Phase 2: Development of standards and assessment tool**

**Study design:** A descriptive cross-sectional study will be utilised for this phase [14].

**Methodology:** The modified Delphi method will be used in Phase 2 of the study. This research method was developed by the RAND (Research and Development) Corporation in a military setting around 1948, and was initially used for classified research [54]. The Delphi technique can be used in research for different reasons, such as to build consensus on a topic in a situation of uncertainty, or where there is a lack of empirical evidence to make predictions in order to better understand possible future scenarios, and to choose strategies for action, such as with military interventions and education methods/practices [51,54–57].

A Delphi study is therefore ideal to achieve consensus on the standards needed for a tool that can be used for self- and peer-review. Since the Delphi technique is exploratory in nature, it enables the best management of group dynamics by reaching an agreement among experts while preventing possible derailments due to opposing views. This technique achieves results through consecutive stages of data collection regarding consensus, which usually involves working through three distinctive rounds of expert consultation [51,54–56].

For this study, the Delphi survey will be developed informed by the relevant literature, and the results from Phase 1. It will be administered electronically (using Qualtrics® software [58]; https://pretoria.eu.qualtrics.com/), and will consist of three rounds to ensure good response rates. The Delphi panel will comprise the participants (MPH coordinators) from the academic institutions. The duration for the participants’ response will be exactly one month, from the initial invitation with reminders.

After completion of the first round, an updated survey will be compiled and sent to the panellists, and this will constitute the second round. The updated survey will include the descriptive statistics on the repeated statements (for which there was no consensus) to provide insight on other participants’ responses as they engage with the survey again; but will exclude the statements on which there was consensus in the first round. Depending on the consensus results of the second round, the third round will be instituted [54–56,59].

## Measurements

### Measurement instruments

The measurement instruments will be developed for each phase of the study. The measurement instruments will be available in the three languages (English, French, Portuguese) to enable the participants to choose their most proficient language.

### Phase 1: Exploration of the current status of the academic institutions of public health

An electronic survey using a self-administered questionnaire is being employed for this Phase using the Qualtrics® software [58] (https://pretoria.eu.qualtrics.com/) to measure the study variables shown in Table 2. The section headings will include; characteristics of the academic institutions, quality assurance of teaching and learning methods strategies, MPH programme design and curriculum renewal strategies, professional development of academic faculty and assessing the need and acceptability of a regional accreditation body for the academic institutions of public health. Since the study will not use a validated questionnaire, Cronbach’s alpha will be calculated to ensure internal consistency, an objective measure of reliability used to overcome measurement error [60].

**Table 2:** Phase 1 study variables to be measured. (Adapted from BME WFME 2020 [64] Standards and HPCSA Guidelines [38]).

SECTION 1: MPH PROGRAMME DESIGN
• Relation to institution’s mission and planning • Needs of students and other stakeholders • Intellectual credibility • Coherence and articulation • Characteristics and needs of professional and vocational education • Learning materials development
**SECTION 2: STUDENT RECRUITMENT, ADMISSION AND SELECTION**
• Recruitment, admission and selection • Legislative framework • Widening of access; Equity • Assumptions of learning • Professional needs • Capacity of the programme to offer quality education
**SECTION 3: ACADEMIC FACULTY STAFFING AND DEVELOPMENT**
• Qualifications • Teaching experience • Assessment competence and feedback • Research profile • Staff development • Size and seniority • Full-time and part-time staff
**SECTION 4: TEACHING AND LEARNING STRATEGIES**
• Importance of promotion of student learning • Institutional type, mode(s) of delivery and student composition • Appropriate teaching and learning methods • Quality assurance: curriculum renewal plan • Targets, implementation plans, and ways to monitor, evaluate impact, and effect improvement
**SECTION 5: STUDENT ASSESSMENT POLICIES AND PROCEDURES**
• Internal assessment policies and procedures • Internal and external moderation • Monitoring of student progress • Validity and reliability of assessment
**SECTION 6: INFRASTRUCTURE AND LIBRARY RESOURCES**
• IT infrastructure and training • Size and scope of library resources • Integration of library resources into curriculum • Management and maintenance of library resources **•** Library support and access to students
**SECTION 7: PROGRAMME ADMINISTRATIVE SERVICES**
• Provision of information • Identifying non-active and at-risk students • Dealing with the needs of a diverse student population • Ensuring the integrity of certification
**SECTION 8: PROGRAMME COORDINATION**
• Mandate and responsibilities of the programme coordinator(s) • Student input and participation • Implementation of policies for ensuring the integrity of certification
**SECTION 9: ACADEMIC DEVELOPMENT FOR STUDENT SUCCESS**
• Student and staff development • Curriculum development (and renewal) • Additional student academic support
**SECTION 10: STUDENT ASSESSMENT PRACTICES**
• Integral part of teaching and learning practices • Internal (or external) assessment • Internal and external moderation • Reliability, rigour and security
**SECTION 11: ACCREDITATION SYSTEM FOR ACADEMIC INSTITUTIONS OF PUBLIC HEALTH IN AFRICA**
• Interest and need for an accreditation system • Suggested accreditation body • Intervals of accreditation

The necessary privacy and safety requirements will continually be ensured. All the research data will be recorded and stored securely for analysis purposes. A password-protected database will be used, which will be backed-up regularly, and will only be accessible to the primary researcher and the research supervisors. Ethical approval has been obtained (Ref. no. 2352021) from the Faculty of Health Sciences Research Ethics Committee at the University of Pretoria [61–63].

### Phase 2: Development of standards and assessment tool

The measurement instrument will comprise an electronic self-administered questionnaire using Qualtrics® software [58] (https://pretoria.eu.qualtrics.com/), to be administered to the participants utilising the Delphi technique. The results of Phase 1 will be summarised as possible standards for consideration and these draft standards will form the basis of the Delphi survey. The five-point Likert scale online surveys will be used to obtain data from the participants. After completion of the first round, an updated survey will be compiled and sent to the panellists, and this will constitute the second round. The updated survey will include the descriptive statistics on the repeated statements (for which there was no consensus) to provide insight on other participants’ responses as they engage with the survey again; but will exclude the statements (greyed-out) on which there was consensus in the first round. Depending on the consensus results of the second round, the third round will be instituted. The proportions for each of the Delphi survey items will be derived. Consensus will be defined as the combination of participants who *agree* and *strongly agree* to the statements. Non-consensus will be defined as a combination of *disagree* and *strongly disagree* as well as *neutral/ do not know* responses [54–57,59,65].

### Data management and analysis plan

The collected data will only be accessible to the principal researcher and the research supervisors. The data will be kept private and treated with the strictest confidence. The data will be downloaded from the Qualtrics® platform [58] into a password-protected database (with continuous backing up), and will be exported to Stata version 18 for analysis after each round.

All statistical analyses will be conducted by the principal researcher and consultation with a biostatistician will be sought, if required. The data analysis will be performed in accordance with the three phases of the study.

###  Phase 1: Exploration of the current status of the academic institutions of public health

#### Data analysis

##### Data analyses will comprise the following

**Quantitative data analysis:** Firstly, the data will be declared as survey design (command: *svyset*), since the sampling method for this phase will be multi-stage. This will include stratification and sampling weights (probability weights or pweights) will be necessary as the participants may have different probabilities of selection where others may be oversampled due to the uneven distribution of the academic institutions within the sub-regions [14,66].

Descriptive statistics (means, modes and range [including interquartile range: IQR]) will be used to summarise categorical variables, and frequency distributions (proportions and percentages) [14,59]. Reliability and coherence between questionnaire items will be tested using the Cronbach alpha coefficient [60]. Factor analysis and sensitivity analysis of the questionnaire items will be conducted. Logistic regression will be performed to determine the associations between the study variables [67,68].

All quantitative data analyses will be done using STATA version 18 (Stata Corp., College Station, TX, USA) [69].

**Qualitative data analysis:** Thematic analysis of the qualitative data derived from open-ended questions will be done using ATLAS.ti, in accordance with the study objectives. The responses will be categorised into predetermined themes based on the sections of the questionnaire [51].

##### Phase 2: Development of standards and assessment tool

###### Data Analysis Plan.

Data analyses will comprise the following:

**Quantitative data analysis:** Descriptive statistics (means, modes and range [including interquartile range: IQR]) will be used to summarise categorical variables, and frequency distributions (proportions and percentages) will be described for participants’ responses measured on the five-point Likert scale (“strongly agree”, “agree”, “neutral/ don’t know”, “disagree”, “strongly disagree”) [14,54,56,59]. Reliability and coherence between questionnaire items will be tested using the Cronbach alpha coefficient. Logistic regression will be performed to determine the associations between the study variables [67,68]. All quantitative data analyses will be done using STATA version 18 (Stata Corp., College Station, TX, USA) [69].

**Qualitative data analysis:** Thematic analysis of the qualitative data derived from open-ended questions will be done using ATLAS.ti, in accordance with the study objectives. The responses will be categorised into predetermined themes based on the sections of the questionnaire [51].

##### Error minimising processes

Every research undertaking has an inherent risk of bias. Selection bias will be minimised through using the total population sampling method [53] where the entire study population will be included in the study. Information bias will be minimised through the upholding of the ethical principles to reassure the study participants of anonymity, privacy and confidentiality of the information provided. Since the study will not use a validated questionnaire, Cronbach’s alpha will be calculated to ensure internal consistency, an objective measure of reliability used to overcome measurement error [60].

Application of the criteria for a good quality Delphi study: specifying the planned number of rounds, specifying the stopping criteria, repeatability criteria used for the selection of study participants, and criteria used to drop off items after each specified round [54,59].

The master questionnaire will be in English and translated using the inbuilt function of Qualtrics® software [58] (https://pretoria.eu.qualtrics.com/). The translation will be validated by the French and Portuguese language experts from the Department of Ancient and Modern Languages and Culture in the Faculty of Humanities, at the University of Pretoria or translation service providers. Various techniques will be used for qualitative data management and analysis to ensure quality criteria of credibility, transferability, dependability and confirmability. These include, (1) iterative data analysis through continuous examining of the data based on the themes and sub-themes emerging from the analysis, (2) audit trail (record-keeping of all the research processes and amendments, if any), and (3) peer debriefing to discuss the process and findings with experts and peers [51,59].

##### Ethical considerations

Ethical approval to conduct the study was obtained from the Faculty of Health Sciences Research Ethics Committee at the University of Pretoria (Ref. no. 2352021). The ethical principles for biomedical research (autonomy, beneficence, non-maleficence and distributive justice) will be upheld throughout the tenure of the research project. The participant information and informed consent document will be administered electronically via the Qualtrics platform, before participants can commence with responses. The document provides details on the nature of the study and what is expected from the study participants (including, the duration), their right to refuse or withdraw without any penalty, as well as the contact details of the Research Ethics Committee [61–63].

The participants’ (institutional) details will be de-identified to maintain privacy and confidentiality. There are no anticipated risks during the study. Although the study benefits may not be direct for the research participants, the study findings will benefit the MPH programmes and the institutions within which they work, for the overall benefit of capacity development and quality assurance of the MPH programme as well as the overall quality and training of public health education within the African region. The research stakeholders will be informed of the research findings [61–63].

All the data will be managed by the primary researcher and stored on a password-protected database with backup after downloading from Qualtrics® platform [58]; and only accessible to the research team. All the research data will be stored at the School of Health Systems and Public in the Faculty of Health Sciences for 10 years in accordance with the University of Pretoria Data Management Policy.

## Discussion

### Dissemination plans

The findings of the study will be disseminated through various platforms. The Phase 1 and 2 findings will be submitted as part of a thesis in partial fulfilment of the Doctor of Philosophy in Public Health (PhD Public Health) degree at the School of Health Systems and Public Health in the Faculty of Health Sciences at the University of Pretoria, South Africa.

The developed assessment tool after Phase 2 will be foundational and piloted among willing participants (institutions) and will comprise Phase 3 of the study (post-PhD).

All the study findings will be shared with all the relevant stakeholders, including the study participants through ASPHA as well as the Council for Higher Education (or equivalent). Journal manuscripts will also be submitted to accredited peer-reviewed journals.

The findings will also be presented at national conferences (SAAHE and PHASA), regional (ASPHA) and international conferences (WCPH). The proposed study has been presented at the 17^th^ World Congress on Public Health (WCPH) in Rome, Italy in May 2023 [70].

## Conclusion

The study findings will provide evidence to inform the relevant decision-makers regarding the need for an accreditation system for the MPH programme in Africa. In addition, they will foster long-term networks and partnerships; and further inform the curriculum reform of the academic institutions of public health in Africa to appropriately respond and align to the health needs within the African region in pursuit of realising the United Nations 2030 Agenda of Sustainable Development Goals.

## References

[1] World Health Organization. Frequently Asked Questions Geneva, Switzerland: World Health Organization; 2024 [cited 2025]. Available from: https://www.who.int

[2] World Health Organization. Sustainable Development Goals: Sustainable Development Knowledge Platform Geneva, Switzerland: World Health Organization (WHO); 2023 [cited 2023]. Available from: https://sustainabledevelopment.un.org/?menu=1300

[3] Rispel LC, Blaauw D, Ditlopo P, White J. Human Resources for Health and Universal Health Coverage: Progress, Complexities and Contestations. South African Health Review. 2018(1):13–21.

[4] Matsoso M, Strachan B. Human Resources for Health for South Africa: HRH Strategy for the Health Sector 2012/13 - 2016/17. South African Health Review. 2011;2011(1):49–58.

[5] Dlungwane T, Voce A, Searle R, Stevens F. Master of Public Health programmes in South Africa: issues and challenges. Public Health Rev. 2017;38:5. doi: 10.1186/s40985-017-0052-9 29450077 PMC5810082

[6] Tucker JM, Chalkidou K, Pillay Y. Establishing the NHI Service Benefits Framework: lessons learnt and stakeholder engagement. In: Moeti T, Padarath A, editors. South African Health Review. 22nd ed. Durban, South Africa: Health Systems Trust. 2019. p. 43–53.

[7] Zweigenthal V, London L, Pick W, editor. The contribution of specialist training programmes to the development of a public health workforce in South Africa. South Africa: Health Systems Trust; 2016.

[8] Brownson RC, Samet JM, Bensyl DM. Applied epidemiology and public health: are we training the future generations appropriately?. Ann Epidemiol. 2017;27(2):77–82. doi: 10.1016/j.annepidem.2016.12.002 28038933 PMC5578705

[9] Erwin PC, Brownson RC. Macro Trends and the Future of Public Health Practice. Annu Rev Public Health. 2017;38:393–412. doi: 10.1146/annurev-publhealth-031816-044224 27992728

[10] Baker PRA, Dingle K, Dunne MP. Future of Public Health Training: What Are the Challenges? What Might the Solutions Look Like?. Asia Pac J Public Health. 2018;:1010539518810555. doi: 10.1177/1010539518810555 30444136

[11] Opare A. Harmonizing core competencies for Master of Public Health (MPH) programmes in Africa. 2019 ASPHA Conference. Kampala, Uganda. 2019.

[12] World Health Organization. Global Health Workforce Network. Geneva, Switzerland: World Health Organization (WHO), 2017.

[13] World Health Organization. Global health workforce network: education hub. 2021. https://www.who.int/health-topics/health-workforce#tab=tab_1

[14] Ehrlich R, Joubert G. Epidemiology: A Research Manual for South Africa. 3rd ed. South Africa: Oxford University Press: Southern Africa. 2014.

[15] Thorpe A, Griffiths S, Jewell T, Adshead F. The three domains of public health: an internationally relevant basis for public health education?. Public Health. 2008;122(2):201–10. doi: 10.1016/j.puhe.2007.05.013 17889089 PMC7111666

[16] Brownson RC, Samet JM, Chavez GF, Davies MM, Galea S, Hiatt RA, et al. Charting a future for epidemiologic training. Ann Epidemiol. 2015;25(6):458–65. doi: 10.1016/j.annepidem.2015.03.002 25976024 PMC4646613

[17] Association of Schools of Public Health in Africa. ASPHA Mission, Vision, Objectives. 2024 [cited 2023]. Available from: https://asphaafrica.net/

[18] Birt CA, Foldspang A. The Developing Role of Systems of Competences in Public Health Education and Practice. Public Health Rev. 2011;33(1):134–47. doi: 10.1007/bf03391624

[19] Moser JM. Core academic competencies for master of public health students: one health department practitioner’s perspective. Am J Public Health. 2008;98(9):1559–61. doi: 10.2105/AJPH.2007.117234 18633096 PMC2509593

[20] Negandhi H, Negandhi P, Tiwari R, Sharma A, Zodpey S, Kulatilaka H, et al. Developing core competencies for monitoring and evaluation tracks in South Asian MPH programs. BMC Med Educ. 2015;15:126. doi: 10.1186/s12909-015-0403-5 26238573 PMC4523996

[21] Frenk J, Chen L, Bhutta ZA, Cohen J, Crisp N, Evans T, et al. Health professionals for a new century: transforming education to strengthen health systems in an interdependent world. Lancet. 2010;376(9756):1923–58. doi: 10.1016/S0140-6736(10)61854-5 21112623

[22] Zweigenthal V, Patrick SM. Project start-up meeting - core competencies and career paths of Master of Public Health (MPH) graduates in South Africa: A comparative study. School of Public Health, University of the Witwatersrand. 2019.

[23] Association of Schools of Public Health in the Europe Region. ASPHER: Mission, Functions and Objectives. 2023. Available from: http://www.aspher.org

[24] Leslie K, Moore J, Robertson C, Bilton D, Hirschkorn K, Langelier MH, et al. Regulating health professional scopes of practice: comparing institutional arrangements and approaches in the US, Canada, Australia and the UK. Hum Resour Health. 2021;19(1):15. doi: 10.1186/s12960-020-00550-3 33509209 PMC7841037

[25] Mansholt H, Czabanowska K, Otok R, de Nooijer J. Professionalization of public health – an exploratory case study. SEEJPH. 2020;:1–16. doi: 10.4119/seejph-3845

[26] Hongoro C, McPake B. How to bridge the gap in human resources for health. Lancet. 2004;364(9443):1451–6. 15488222 10.1016/S0140-6736(04)17229-2

[27] World Health Organization. Everybody’s business: strengthening health systems to improve health outcomes: WHO’s framework for action. Geneva, Switzerland: World Health Organization. 2007.

[28] Kieny MP, Bekedam H, Dovlo D, Fitzgerald J, Habicht J, Harrison G, et al. Strengthening health systems for universal health coverage and sustainable development. Bull World Health Organ. 2017;95(7):537–9. doi: 10.2471/BLT.16.187476 28670019 PMC5487973

[29] World Health Organization W. WHO | Global Health Workforce Network: World Health Organization; 2022 [cited 2023]. Available from: https://www.who.int/teams/health-workforce/network

[30] Zodpey SP, Evashwick CJ, Grivna M, Harrison RA, Finnegan JR. Editorial: Educating the Global Workforce for Public Health. Front Public Health. 2018;5:364. doi: 10.3389/fpubh.2017.00364 29404315 PMC5786571

[31] White F. The Imperative of Public Health Education: A Global Perspective. Med Princ Pract. 2013;22(6):515–29. doi: 10.1159/000354198 23969636 PMC5586806

[32] Chen C, Buch E, Wassermann T, Frehywot S, Mullan F, Omaswa F, et al. A survey of Sub-Saharan African medical schools. Hum Resour Health. 2012;10:4. doi: 10.1186/1478-4491-10-4 22364206 PMC3311571

[33] Peik SM, Mohan KM, Baba T, Donadel M, Labruto A, Loh LC. Comparison of public health and preventive medicine physician specialty training in six countries: Identifying challenges and opportunities. Med Teach. 2016;38(11):1146–51. doi: 10.3109/0142159X.2016.1170784 27093229

[34] Tulchinsky TH, Goodman J. The role of schools of public health in capacity building. J Public Health (Oxf). 2012;34(3):462–4. doi: 10.1093/pubmed/fds045 22722092

[35] Evans D. The role of schools of public health: learning from history, looking to the future. J Public Health (Oxf). 2009;31(3):446–50. doi: 10.1093/pubmed/fdp065 19574273

[36] Ijsselmuiden CB, Nchinda TC, Duale S, Tumwesigye NM, Serwadda D. Mapping Africa’s advanced public health education capacity: the AfriHealth project. Bull World Health Organ. 2007;85(12):914–22. doi: 10.2471/blt.07.045526 18278250 PMC2636289

[37] University Research Co. LLC. USAID: Applying Science to Strengthen and Improve Systems (ASSIST) Project. 2020. https://www.urc-chs.com/projects/usaid-applying-science-strengthen-and-improve-systems-assist-project

[38] Health Professions Council of South Africa. Guidelines for Evaluation and Accreditation of Higher Education and Training Institutions. Pretoria, South Africa: Health Professions Council of South Africa. 2016.

[39] Biomedcentral Medicine. Health workforce: Accreditation of education and regulation of practice 2020 [cited 2023;}. Available from: https://www.biomedcentral.com/collections/accred

[40] Burdick W, Dhillon I. Ensuring quality of health workforce education and practice: strengthening roles of accreditation and regulatory systems. Hum Resour Health. 2020;18(1):71. doi: 10.1186/s12960-020-00517-4 33076909 PMC7572237

[41] Otok R, Levin I, Sitko S, Flahault A. European Accreditation of Public Health Education. Public Health Rev. 2011;33(1):30–8. doi: 10.1007/bf03391619

[42] Council on Education for Public Health. Programmatic Accrediting Organization. Silver Spring, Maryland, USA: Council on Education for Public Health (CEPH). 2020.

[43] Professional Board for Emergency Care H. Form 332: Guidelines for the completion of the portfolio for institutions intending to offer the higher certificate, diploma and/or bachelors degree in emergency medical care. Pretoria, South Africa: Health Professions Council of South Africa. 2017.

[44] Pillay-van Wyk V, Msemburi W, Laubscher R, Dorrington RE, Groenewald P, Glass T, et al. Mortality trends and differentials in South Africa from 1997 to 2012: second National Burden of Disease Study. Lancet Glob Health. 2016;4(9):e642-53. doi: 10.1016/S2214-109X(16)30113-9 27539806

[45] Association of Schools of Public Health in Africa. Communique from 2019 ASPHA Conference. Theme: Universal health coverage in Africa: The role of public health workforce. Kampala, Uganda: Association of Schools of Public Health in Africa (ASPHA). 2019.

[46] 16th World Congress on Public Health. Public Health for the Future of Humanity: Analysis, Advocacy and Action. 16th World Congress on Public Health (WCPH); 12-16 October 2020; Rome, Italy. 2020.

[47] EUPHA Health Workforce Research Section Conference. How to make a future health workforce happen? Policy, practice and people. EUPHA Health Workforce Research Section Midterm Virtual Conference; 2020; Babes-Bolyai University, Romania. 2020.

[48] Nathan N. The public health workforce development in the European region. In: Kampala, Uganda, 2019.

[49] Otok R, Czabanowska K, Foldspang A. Public health educational comprehensiveness: The strategic rationale in establishing networks among schools of public health. Scand J Public Health. 2017;45(7):720–2. doi: 10.1177/1403494817738498 29162017

[50] Creswell JD, Plano Clark VL. Designing and conducting mixed methods research. 3rd ed. SAGE Publications, Inc. 2017.

[51] Creswell JW, Creswell JD. Research design: qualitative, quantitative, and mixed methods approaches. 5 ed. Thousand Oaks, California: SAGE Publications, Inc. 2018.

[52] World Health Organization. WHO African Region. Geneva, Switzerland: World Health Organization (WHO); 2023. Available from: https://www.afro.who.int/countries

[53] Lavrakas P. Encyclopedia of Survey Research Methods: SAGE Publications; 2008 [cited 2025]. Available from: http://dissertation.laerd.com/total-population-sampling.php

[54] Mariotti D, McAuliffe GJ, Grothaus T, West-Olatunji C, Snow KC. Towards a New Profession: Counselor Professional Identity in Italy. A Delphi Study. Int J Adv Counselling. 2019;41(4):561–79. doi: 10.1007/s10447-019-09376-8

[55] de Villiers MR, de Villiers PJT, Kent AP. The Delphi technique in health sciences education research. Med Teach. 2005;27(7):639–43. doi: 10.1080/13611260500069947 16332558

[56] Massaroli AMJ, Lino MM, Spenassato D, Massaroli R. The delphi method as a methodological framework for research in nursing. Texto Contexto Enferm. 2017;26(4).

[57] Okoli C, Pawlowski SD. The Delphi method as a research tool: an example, design considerations and applications. Information & Management. 2004;42(1):15–29. doi: 10.1016/j.im.2003.11.002

[58] Qualtrics Software Package. Provo, Utah, USA: Qualtrics. 2023.

[59] Slade SC, Dionne CE, Underwood M, Buchbinder R. Standardised method for reporting exercise programmes: protocol for a modified Delphi study. BMJ Open. 2014;4(12):e006682. doi: 10.1136/bmjopen-2014-006682 25550297 PMC4281530

[60] Tavakol M, Dennick R. Making sense of Cronbach’s alpha. Int J Med Educ. 2011;2:53–5. doi: 10.5116/ijme.4dfb.8dfd 28029643 PMC4205511

[61] National Department of Health. Ethics in Health Research: Principles, Processes and Structures. Pretoria: National Department of Health, Republic of South Africa. 2015.

[62] The National Commission for the Protection of Human Subjects of Biomedical and Behavioral Research. The Belmont Report: Ethical Principles and Guidelines for the Protection of Human Subject of Research. Washington, DC, USA. 1979. 25951677

[63] World Medical Association. World Medical Association Declaration of Helsinki: Ethical Principles for Medical Research Involving Human Subjects. 2013. 10.1001/jama.2013.281053 24141714

[64] World Federation for Medical Education WFME. Basic medical education WFME global standards for quality improvement: The 2020 revision. WFME. 2020.

[65] Hasson F, Keeney S, McKenna H. Research guidelines for the Delphi survey technique. J Adv Nurs. 2000;32(4):1008–15. doi: 10.1046/j.1365-2648.2000.t01-1-01567.x 11095242

[66] Williams R. Analyzing complex survey data: some key issues to be aware of. University of Notre Dame. 2020.

[67] Hosmer DW, Lemeshow S, Sturdivant RX. Applied logistic regression. 3 ed. Hoboken, New Jersey: Wiley. 2013.

[68] Vittinghoff E, Glidden DV, Shiboski SC, McCulloch CE. Regression methods in biostatistics: linear, logistic, survival, and repeated measures models. 2 ed. New York: Springer. 2012.

[69] Stata: Software for Statistics and Data Science. 18 ed. College Station, Texas; USA: StataCorp. 2024.

[70] 17th World Congress on Public Health (WCPH). A World in Turmoil: Opportunities to Focus on the Public's Health. 02-06 May 2023; Rome, Italy. 2023.

